# Occurrence of mitochondrial CO1 pseudogenes in *Neocalanus plumchrus* (Crustacea: Copepoda): Hybridization indicated by recombined nuclear mitochondrial pseudogenes

**DOI:** 10.1371/journal.pone.0172710

**Published:** 2017-02-23

**Authors:** Ryuji J. Machida, Ya-Ying Lin

**Affiliations:** Biodiversity Research Center, Academia Sinica, Nankang, Taipei, Taiwan; Stazione Zoologica Anton Dohrn, ITALY

## Abstract

A portion of the mitochondrial cytochrome *c* oxidase I gene was sequenced using both genomic DNA and complement DNA from three planktonic copepod *Neocalanus* species (*N*. *cristatus*, *N*. *plumchrus*, and *N*. *flemingeri*). Small but critical sequence differences in CO1 were observed between gDNA and cDNA from *N*. *plumchrus*. Furthermore, careful observation revealed the presence of recombination between sequences in gDNA from *N*. *plumchrus*. Moreover, a chimera of the *N*. *cristatus* and *N*. *plumchrus* sequences was obtained from *N*. *plumchrus* gDNA. The observed phenomena can be best explained by the preferential amplification of the nuclear mitochondrial pseudogenes from gDNA of *N*. *plumchrus*. Two conclusions can be drawn from the observations. First, nuclear mitochondrial pseudogenes are pervasive in *N*. *plumchrus*. Second, a mating between a female *N*. *cristatus* and a male *N*. *plumchrus* produced viable offspring, which further backcrossed to a *N*. *plumchrus* individual. These observations not only demonstrate intriguing mating behavior in these species, but also emphasize the importance of careful interpretation of species marker sequences amplified from gDNA.

## Introduction

A growing body of literature is documenting the presence of nuclear copies of mitochondrial sequences, known as nuclear mitochondrial pseudogenes (reviewed in Bensasson et al. 2001[1]). Occurrences of the nuclear mitochondrial pseudogenes are observed in various taxonomic groups in Eukaryotes, and Crustaceans are no exception [2–7]. The paralogous nature of nuclear mitochondrial pseudogenes have profound implications for studies which use mitochondrial DNA sequence as a genetic marker, including studies in the fields of phylogeny, phylogeography, species identification, metagenetics and metabarcoding.

*Neocalanus* copepods are some of the most dominant pelagic copepods in the Pacific [8–10], and certain species of this genus undergo extensive ontogenetic vertical migrations, residing and growing in the euphotic layer from winter to summer, and descending to the meso- and bathypelagic layers from summer to winter (or autumn) for maturation and spawning [8]. During the course of a phylogenetic study of *Neocalanus* copepods [11], we repeatedly obtained sequences of unknown identity, with many polymorphic sites (double peaks) observed in sequence electropherograms. By cloning those PCR products, we detected several haplotypes within single organisms.

In this study we compared the patterns of sequence variations between genomic DNA (gDNA) and complement DNA (cDNA). Genomic DNA is composed of not only mitochondrial but also nuclear chromosomal DNA, which directly extracted from individuals. In contrast, cDNA is reverse transcribed DNA, which synthesized from extracted RNA as template. Therefore, sequences obtained from gDNA include not only active gene sequences, but also non-active pseudogene sequences. In contrast, sequences obtained from cDNA analyses are only actively transcribed gene sequences. From the comparative analyses of gDNA and cDNA, we detected multiple nuclear mitochondria pseudogenes in *N*. *plumchrus* and evidence for a recombination event between these pseudogenes. One pseudogene was a chimera of sequences from *N*. *plumchrus* and *N*. *cristatus*, which demonstrated the production of viable hybrid offspring from a mating between the two species.

## Materials and methods

### Animal samples

Specimens of *Neocalanus* species (*N*. *cristatus*, *N*. *plumchrus*, and *N*. *flemingeri*) were collected off the Pacific coast of southeastern Hokkaido, Japan, using a Norpac net (North Pacific Standard Plankton Net). Specimens were either immediately preserved in RNAlater (Ambion Inc.) or brought back to laboratory alive. An additional sample was also collected off the Pacific coast of Tohoku, northern Japan. No specific permissions were required for these activities and work did not involve endangered or protected species.

### RNA and DNA extraction

At least 3 individuals were examined from each species. Bodies of each individual were divided into two parts using sterilized scissors and served for gDNA and messenger RNA (mRNA) extraction. Although only gDNA and not mRNA was extracted from a *N*. *plumchrus* individual caught off the Pacific coast of Tohoku (Table 1), we included this organism in the analysis as it contains a putative chimera sequence (discussed later).

**Table 1:** Summary of mitochondrial cytochrome *c* oxidase I gene sequences from three *Neocalanus* species.

*Neocalanus* species	Individual number	gDNA			cDNA		
		Number of clones sequenced	Number of substitutions Non-synonymous / synonymous	Accession number (INSDC)	Number of clones sequenced	Number of substitutions Non-synonymous / synonymous	Accession number (INSDC)
*N*. *cristatus*	381	5	3 / 3	AB099151-AB099155	7	5 / 1	AB099175-AB099181
	382	8	7 / 8	AB099156-AB099163	5	0 / 0	AB099182-AB099186
	383	7	4 / 2	AB099164-AB099170	7	1 / 3	AB099187-AB099193
	384	4	8 / 27	AB099171-AB099174	7	2 / 1	AB099194-AB099200
*N*. *flemingeri*	387	8	8 / 28	AB099201-AB099208	6	0 / 1	AB099221-AB099226
	388	8	6 / 4	AB099209-AB099216	8	3 / 2	AB099227-AB099234
	389	4	1 / 6	AB099217-AB099220	4	1 / 0	AB099235-AB099238
*N*. *plumchrus*	407	6	28 / 61	AB099239-AB099244	6	6 / 0	AB099267-AB099272
	408	8	14 / 96	AB099245-AB099252	6	3 / 3	AB099273-AB099278
	409	8	29 / 149	AB099253-AB099260	7	6 / 1	AB099279-AB099285
	168	6	7 / 180	AB099261-AB099266			

Total gDNA was extracted from a portion of each individual using a DNeasy (Qiagen Inc.) kit, in accordance with the manufacturer’s protocol. Messenger RNA was selectively extracted from the other half of each individual by oligo-(dT) cellulose chromatography using the QuickPrep Micro mRNA Purification Kit (Amersham Biosciences Inc.).

### Amplification from gDNA

**Fig 1:**
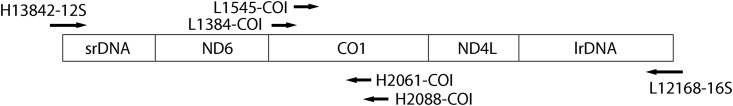
A portion of the mitochondrial genome amplified in the present study. Primers used for the amplification were indicated as arrows. Refer to the text for the primer sequences.

Mitochondrial DNA (ca. 4 kbp) was amplified by long PCR with the H13842-12S (5′-TGT GCC AGC ASC TGC GGT TAK AC-3′) and L12168-16S (5′-CGT CGA TTT KAA CTC AAA TCA TGT-3′) primers (Fig 1 [11,12]). Thermal condition of the long PCR was as follows. Shuttle long PCR thermal profile: 10 seconds denaturation at 98°C, followed by annealing and extension combined at the same temperature (68°C) for 6 minutes. Temperature for the annealing and extension was decreased by– 0.5°C each cycle during the first 16 cycles from 68°C to 60°C, followed by an additional 21 cycles with fixed temperature (60°C). The reactions contain the following reagents: a total of 25 μl volume with 12.75 μl of sterile, distilled H_2_O, 2.5 μl of 10 X buffer, 4 μl of dNTPs (2.5 mM each), 1.0 μl of each primer (5 μM), 0.25 μl of 1.25 units of LA Taq (Takara Bio Inc.), and 1.0 μl of template. The long PCR products were electrophoresed on a 1.0% L 03 agarose gel (Takara Bio Inc.), which was subsequently stained with ethidium bromide for band characterization under ultraviolet transillumination.

Next, nested PCR using a 50-fold dilution of the long PCR products as a templates was performed to amplify a segment of the mitochondrial cytochrome *c* oxidase I (CO1) gene. L1384-COI (5′-GGT CAT GTA ATC ATA AAG ATA TTG G-3′) and H2088-COI (5′-GAT GTG KGA GAT TAG TCC GAA-3′) primers were used for the *N*. *plumchrus*, and L1384-COI (5′-GGT CAT GTA ATC ATA AAG ATA TTG G-3′) and H2061-COI (5′-GGA ATT AGA ATG TAT GCC TC-3′) primers were used for *N*. *cristatus* and *N*. *flemingeri* [11,12]. The expected length of the PCR products was 704 and 677 bp for *N*. *plumchrus* and *N*. *flemingeri*, respectively. The thermal condition of the PCR was as follows: 30 cycles of 5 second denaturation at 94°C, followed by 5 second annealing at 50°C, and 60 second extension at 72°C. The reaction contained the following reagents: a total of 15-μl reaction volume with 9.8 μl of sterile, distilled H_2_O, 1.5 μl of 10 X buffer, 1.0 μl of dNTPs (2.5 mM each), 1.0 μl of each primer (5 μM), 0.1 μl of 0.25 units of EX *Taq* (Takara Bio Inc.), and 1.0 μl of template. The PCR products were electrophoresed on a 1% L 03 agarose gel (Takara Bio Inc.), which was then stained with ethidium bromide for band characterization under ultraviolet transillumination.

All PCR were performed using a Model 9700 thermal cycler (Applied Biosystems Inc.).

### Reverse transcription of the CO1 gene

First-strand synthesis of cDNA was performed using the 3´-Full Race Core Set (Takara Bio Inc.), in accordance with the manufacturer’s instructions. A 2 μl aliquot of the resulting cDNA was mixed with 33.6 μl of sterile, distilled H_2_O, 5.0 μl of 10 X buffer, 5.0 μl of dNTP (2.5 mM each), 2.0 μl of each primer (5 μM), and 0.4 μl of 0.25 units of EX *Taq* (Takara Bio Inc.), for a 50-μl total reaction volume. L1384-COI and 3sites Adaptor Primer (3´-Full Race Core Set; Takara Bio Inc.) pairs were used. Thermal cycle parameters were the same as stated above for nested PCR, with the sole exception of the extension time, which was increased to 120 seconds. Thirty cycles were performed. Since clear single bands were not observed in PCR products from *N*. *cristatus* and *N*. *plumchrus*, a second round of amplification was carried out in each case, using 1.0 μl of the previous PCR product as a template. L1545-COI (5′-GCT CAT GCW TTT GTC ATG ATT TTT TTT ATG G-3′ [11]) and 3sites Adapter Primer (3´-Full Race Core Set; Takara Bio Inc.) pairs were used in this step. The PCR procedure was the same as that of the first round of amplification.

### Cloning

Prior to cloning, PCR amplicons from gDNA and cDNA were gel-purified on a 1.0% L 03 agarose gel (Takara Bio Inc.). The bands were excised and treated with the Wizard SV Gel and PCR Clean-Up System (Promega Inc.). Purified products were cloned into DH5 α cells using the pGEM-T Easy Vector System (Promega Inc.), following the manufacturer’s instructions. Individual bacterial colonies were picked from the bacterial plate using sterilized toothpicks, and then resuspended in a PCR mix containing SP6 and T7 primers. Reaction concentrations and thermal cycle parameters were the same as those of the nested PCRs (described above), except for the volume, which was scaled down to 10 μl. PCR products with the correctly sized insert were subjected to enzymatic clean up using a Pre-Sequencing kit (USB Inc.).

### Sequencing

Purified PCR products were directly subjected to Sanger sequencing (Applied Biosystems Inc.). Both primers were used for each sequencing reaction, and adenine stretches were confirmed in the case of cDNA analyses. All sequencing reactions were performed according to the manufacturer’s instructions. Labeled fragments were analyzed on an Applied Biosystems 3100 DNA sequencer. Four to eight clones were sequenced for both gDNA and cDNA from each individual (Table 1).

### Phylogenetic analysis

We estimated phylogenetic relationships of the obtained clone sequences using *N*. *tonsus* as the outgroup (INSDC: AB093146). First, the sequences were aligned using the MAFFT server with G-INS-1 [13]. Next, Maximum-likelihood (ML) analysis was performed using the RAxML 7.7.1 server [14] with the following options: -f a -m GTRCATI. The best-score ML tree was selected for examination in the following discussion.

## Results

**Fig 2:**
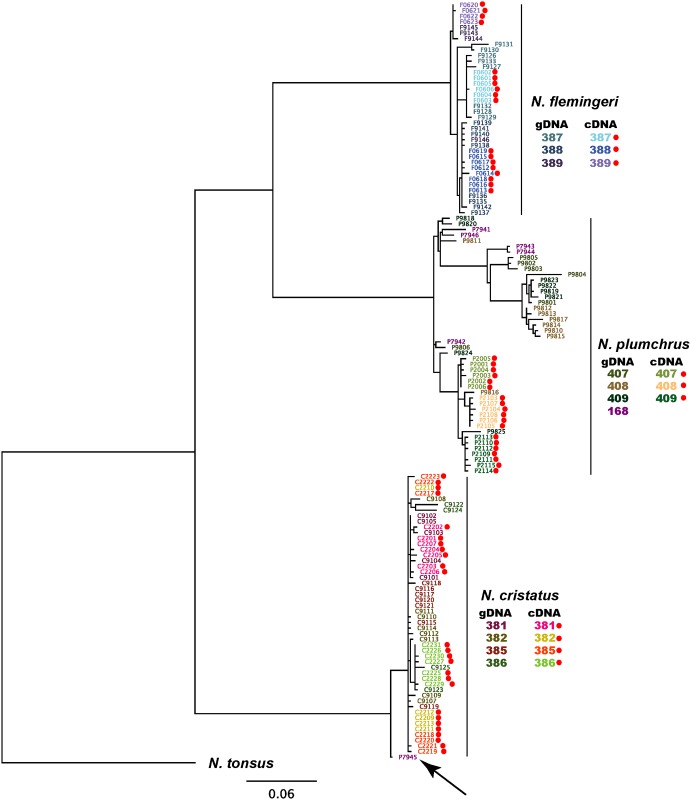
The best-score Maximum Likelihood tree of the mitochondrial cytochrome *c* oxidase I gene clone sequences obtained from *Neocalanus* copepods. Clone number P7945, which was amplified from the genomic DNA of *N*. *plumchrus* but clustered in the clade of *N*. *cristatus*, is indicated by the arrow. Each clone is shown in a different color to enable comparison between individuals and between genomic DNA and complement DNA. Red small circle indicates the clone, which was amplified from complement DNA.

Most of the sequences from different species formed different clades, with three long branches (Fig 2). An exception was clone P7945, which was amplified from the gDNA of *Neocalanus plumchrus* but clustered in the *N*. *cristatus* clade (discussed below). For *N*. *flemingeri* and *N*. *cristatus*, there were very minor sequence differences between clones from gDNA and cDNA, which lead to a flat-top topology. Very small differences were also observed between clone sequences from cDNA of *N*. *plumchrus*. In contrast, the extent of sequence differences varied considerably between clones amplified from gDNA of *N*. *plumchrus*, with a considerable number of synonymous substitutions (Table 1 and Fig 2).

**Fig 3:**
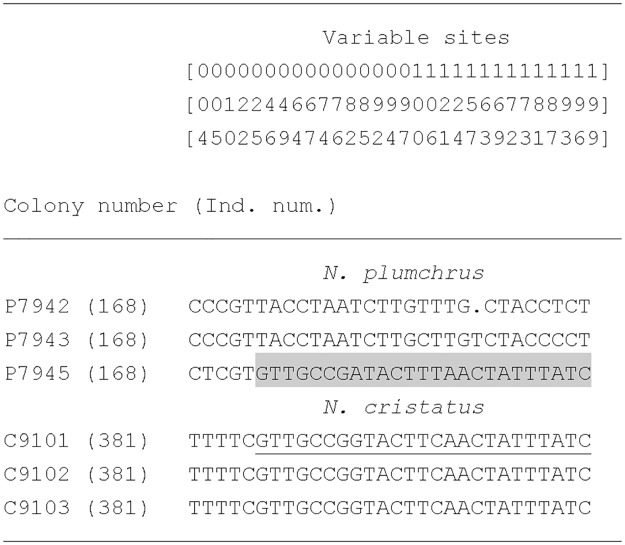
Alignment of mitochondrial cytochrome *c* oxidase I gene sequences from *Neocalanus cristatus* and *N*. *plumchrus* individuals. Only sites with variations in more than two individuals were extracted. Numbers at the top of the figure refer to the 30 variable sites. Clone number P7945 is the sequence that was amplified from genomic DNA of *N*. *plumchrus*, but fell in the *N*. *cristatus* cluster (as shown in Fig 2). The grey box indicates the sequence region in which recombination is believed to have occurred. Underlined letters indicate the putative source segment of the recombination.

One sequence amplified from gDNA of *N*. *plumchrus*, which fell in the *N*. *cristatus* clade (Fig 2: P7945), was aligned with sequences obtained from *N*. *cristatus* and *N*. *plumchrus* (Fig 3). This alignment revealed that the first 25 base pairs of P7945 were identical to the sequence from *N*. *plumchrus*, but the sequence from position 46 to the 3’ end (indicated by the grey box) was similar to that of *N*. *cristatus*.

**Fig 4:**
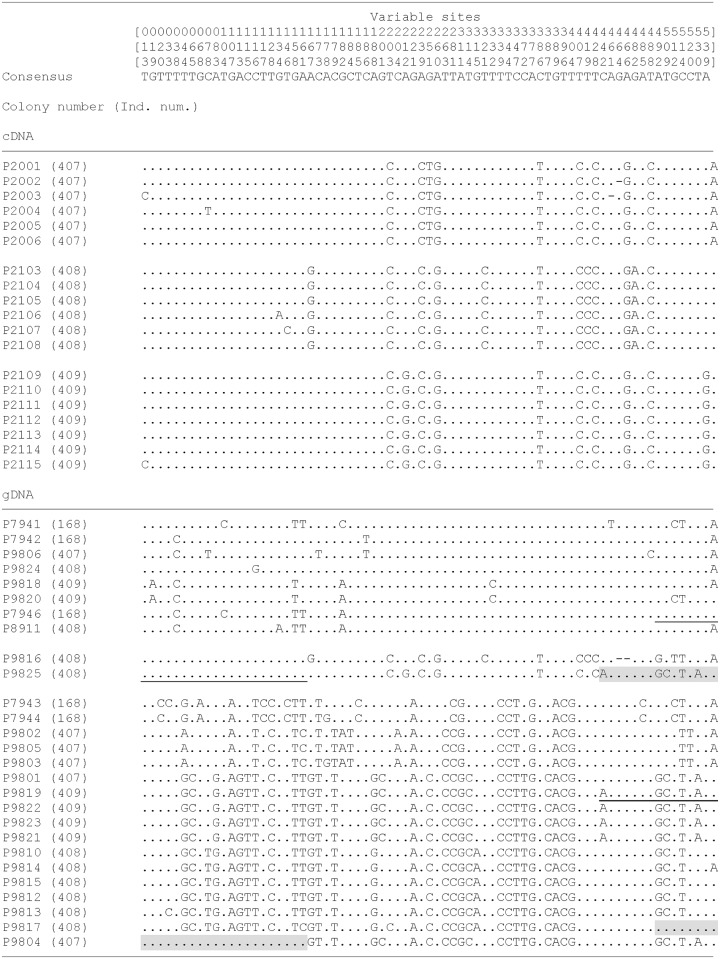
Alignment of all of the mitochondrial cytochrome *c* oxidase I gene sequences from both complement DNA and genomic DNA of *Neocalanus plumchrus*. Only the sites in which variations were observed in more than two individuals were extracted. Numbers at the top of the figure refer to the 72 variable sites. The grey box indicates the region in which recombination is believed to have occurred. Underlined letters indicate the putative source segment of the recombination.

We also aligned all of the sequences from both cDNA and gDNA of *N*. *plumchrus* (Fig 4). Three types of haplotype were obtained from cDNA, which correspond with each individual. In contrast, much higher sequence variations were observed for the sequences obtained from gDNA, which loosely fall into three groups. Careful comparisons of the aligned sequences confirmed that the three regions indicated by grey boxes (Fig 4) were recombinants; underlined letters indicated the putative source segment of the recombination.

## Discussion

In all *Neocalanus* species, sequencing of cDNA clones generally reveals one haplotype for each individual, with putative PCR and cloning miscopies [15]. In contrast, various types of mitochondrial CO1 gene sequence were obtained from gDNA of *N*. *plumchrus* individuals, with a high number of synonymous substitutions (Table 1).

These results observed in the present studies are best explained by the presence of mitochondrial pseudogenes in nuclear genomes because of following reasons. First, we have observed large number of recombined sequences. In general, recombination occurs in the nuclear genome, but not in the mitochondrial genome. Second, we observed only one sequence from cDNA but multiple sequences from gDNA. Differences in nuclear and mitochondrial genetic codes mean that mitochondrial DNA within the nuclear genome is nonfunctional. Moreover, there is only a single promoter for transcription of either strand in the mitochondrial genome, and not individual promoters for each gene, as is observed in nuclear DNA [16]. For these two reasons, it is expected that nuclear mitochondrial pseudogenes will not be transcribed into mRNA [17].

One might suspect that the recombination took place during PCR. However, two lines of evidence indicate that it is very unlikely. First, in general, PCR recombination is considered as a rare event compared to other PCR errors [18], unless the starting template has been seriously damaged [19]. In the present study, four out of 28 cloned sequences exhibited recombination (Figs 3 and 4). Second, we have obtained chimera sequence of *N*. *plumchrus* and *N*. *cristatus* from *N*. *plumchrus* gDNA (Fig 3). If this sequence is created during PCR, template contamination and PCR recombination, both of which are rare events, need to occur simultaneously. Furthermore, we did not observe non-recombined sequence of *N*. *cristatus*, which expected to be observed if template contamination took place. These lines of evidence indicated it is very unlikely that those recombined products were created during the PCR. In general, animal mitochondrial DNA sequences evolve faster than nuclear DNA sequences. As such, once mitochondrial DNA inserts into the nuclear genome, it becomes a ‘fossil’ sequence [20]. Repeated transfer of the mitochondrial DNA sequence to the nuclear genome generates multiple haplotypes with a predominance of silent substitutions.

The genomes of calanoid copepods are very large [21], and there is a strong relationship between the occurrence of nuclear mitochondrial pseudogenes and nuclear genome size [22,23]. In addition, increasing amounts of evidence suggest a high rate of transfer of mitochondrial genes to the nuclear genome [24–31], and pseudogene copies of mitochondrial genes have been observed in a wide range of organisms [32–35], including crustaceans [2–7].

The above lines of observation have important implications for the use of mitochondrial genetic markers in species identification (such as DNA barcoding) and estimation of community diversity (metagenetics). First, most of the sequences from gDNA of *N*. *plumchrus* did not have indels. Therefore, these sequences can be informatically translated into full proteins, which make it impossible to ascertain whether they are pseudogenes or functional protein coding regions from their nucleotide sequences. Second, one organism contained a sequence from a different species (*N*. *cristatus* in this study). Third, we observed prevalent nuclear mitochondrial pseudogene sequences and recombination between some pseudogenes. Several thoughtful discussions regarding avoidance of possible contamination of nuclear mitochondrial pseudogenes for analyses have been previously published [1,5,7]. Furthermore, more and more studies are using massively-parallel sequencers, which can detect rare variants. The results of the present study further demonstrate the importance of careful translation of data obtained from analyses of mitochondrial marker regions amplified from gDNA.

One clone sequence amplified from the gDNA of *N*. *plumchrus* was a chimera sequence of *N*. *plumchrus* and *N*. *cristatus* (clone num. P7945; Figs 2 and 3). Three genetic events need to have occurred to explain this phenomenon. First, hybridization has taken place between a female *N*. *cristatus* individual and a male *N*. *plumchrus* individual, followed by backcrossing to a *N*. *plumchrus* individual. Second, the mitochondrial sequence has been transferred to the nuclear genome. Third, recombination had occurred between the nuclear mitochondrial pseudogenes from *N*. *plumchrus* and *N*. *cristatus*. To our knowledge, while recombination of mitochondrial pseudogenes has been previously reported in human and bird [28,36], our study is the first to detect hybridization based on recombination of mitochondrial pseudogenes. Although, all of the *Neocalanus* species analyzed in the present study constitute a single monophyletic group in the genus [11], body size of *N*. *cristatus* (Female prosome length: 7.74 ± 0.41 mm; [9]) is much longer than that of the other two species (*N*. *flemingeri*: 4.09 ± 0.32 mm, *N*. *plumchrus*: 4.06 ± 0.18 mm; [37,38]). Therefore, the present study has demonstrated hybridization of morphologically dissimilar species (*N*. *cristatus* and *N*. *plumchrus*). One explanation for this unlikely mating is the prolonged reproductive duration observed in *N*. *cristatus* [8,9], which overlaps with that of *N*. *plumchrus*. In contrast, *N*. *plumchrus* and *N*. *flemingeri* exhibit similar body lengths, but have limited mating durations, which do not overlap with each other [10]. To our knowledge, only one other study has demonstrated hybridization of copepods in a natural environment [39]. Together with the present study, these observations indicate that hybridization between species in which mating times overlap is more common than previously thought.
